# The Role of EVs in the Aging Hematopoietic System

**DOI:** 10.1111/acel.70715

**Published:** 2026-09-23

**Authors:** Esra G. Kosebent, Nathalie Havranek, Karoline Kollmann, Kristina Kirschner, Eszter Doma, Kavya Gupta

**Affiliations:** ^1^ School of Cancer Sciences, University of Glasgow Glasgow UK; ^2^ Department of Biological Sciences and Pathobiology Institute of Pharmacology and Toxicology, University of Veterinary Medicine Vienna Vienna Austria; ^3^ Department Internal Medicine, Division of Hematology Mayo Clinic Rochester Minnesota USA; ^4^ Robert and Arlene Kogod Center on Aging, Mayo Clinic Rochester Minnesota USA; ^5^ Department of Biochemistry and Molecular Biology Mayo Clinic Rochester Minnesota USA

**Keywords:** aging, bone marrow microenvironment, extracellular vesicles, rejuvenation

## Abstract

Extracellular vesicles (EVs) are membrane‐enclosed nanoparticles that mediate intercellular communication by transferring proteins, nucleic acids, and lipids. Within the hematopoietic system, EVs orchestrate critical signaling between bone marrow (BM) niche cells and hematopoietic stem and progenitor cells (HSPCs) to regulate quiescence, proliferation, and lineage commitment. During aging, senescence and telomere attrition drive a profound remodeling of EV biogenesis and cargo. Aged EVs, particularly those released from senescent mesenchymal stromal cells (MSCs) and osteoblasts, exhibit altered microRNA profiles and reduced protective antioxidants. These changes perpetuate niche dysfunction, induce senescence in recipient cells, and promote myeloid‐biased hematopoiesis. Importantly, this communication is a selective process governed by vesicle‐intrinsic surface signatures, such as tetraspanins and integrins, which interact with specific receptors on distinct hematopoietic subsets to regulate signaling fidelity. Conversely, EVs from young or pluripotent stem cells deliver rejuvenating signals that can restore aged HSPC function and attenuate inflammaging. Elucidating these mechanisms of selective targeting and stress‐responsive cargo remodeling provides vital insights for developing EV‐based diagnostic tools and rejuvenation strategies to prevent age‐related immune imbalances and hematologic malignancies.

## Graphical abstract

Extracellular vesicles from aged bone marrow carry pro‐inflammatory cargo that drives blood stem cell decline, while youthful vesicles restore or maintain stem cell function. Circulating vesicles therefore represent both a readout of hematopoietic aging and a potential therapeutic route.

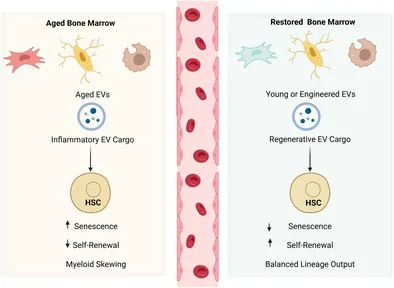

**Abbreviations**
ALIX: ALG‑2‑interacting protein X
ALK5: transforming growth factor β receptor 1
ARCH: age‐related clonal hematopoiesis
BM: bone marrow
BMSC: bone marrow stromal cell
CAR: chimeric antigen receptor
CD: cluster of differentiation
CDK: cyclin dependent kinase
CHIP: clonal hematopoiesis of indeterminate potential
DC: dendritic cell
DRP1: dynamin‐related protein 1
ESCRT: endosomal sorting complex required for transport
EVs: extracellular vesicles
GCA: grancalcin
G‐CSF: granulocyte colony‐stimulating factor
GFP: green fluorescent protein
GMPs: granulocyte–macrophage progenitors
GTP: guanosine triphosphate
HE: hemogenic endothelial
HMOX1: heme oxygenase‐1
HPC: hematopoietic progenitor cell
HSCs: hematopoietic stem cells
HSPCs: hematopoietic stem and progenitor cells
IFN: interferon
IL: interleukin
iPSCs: induced pluripotent stem cells
LAMP: lysosome‐associated membrane glycoprotein
MHC: major histocompatibility complex
miRNA: microRNA
MSCs: mesenchymal stromal cells
MVB: multivesicular body
PCNA: proliferating cell nuclear antigen
PGC1α: peroxisome proliferator‐activated receptor gamma coactivator 1‐alpha
PPARG: peroxisome proliferator‐activated receptor gamma
PRDX: peroxiredoxins
PTH: parathyroid hormone
rGCA: recombinant grancalcin
SASP: senescence‐associated secretory phenotype
sEVs: small extracellular vesicles
SIRT: sirtuin
SNARE: soluble N‐ethylmaleimide‐sensitive‐factor attachment protein receptor
TERT: telomerase reverse transcriptase
TGF: transforming growth factor
TNF: tumor necrosis factor
TPM1: tropomyosin‐1
TRAP: tartrate‐resistant acid phosphatase
TSG: tumor susceptibility gene
VCAM‐1: vascular cell adhesion molecule‐1
VPS4: vacuolar protein sorting 4

## 1 Introduction

**FIGURE 1:**
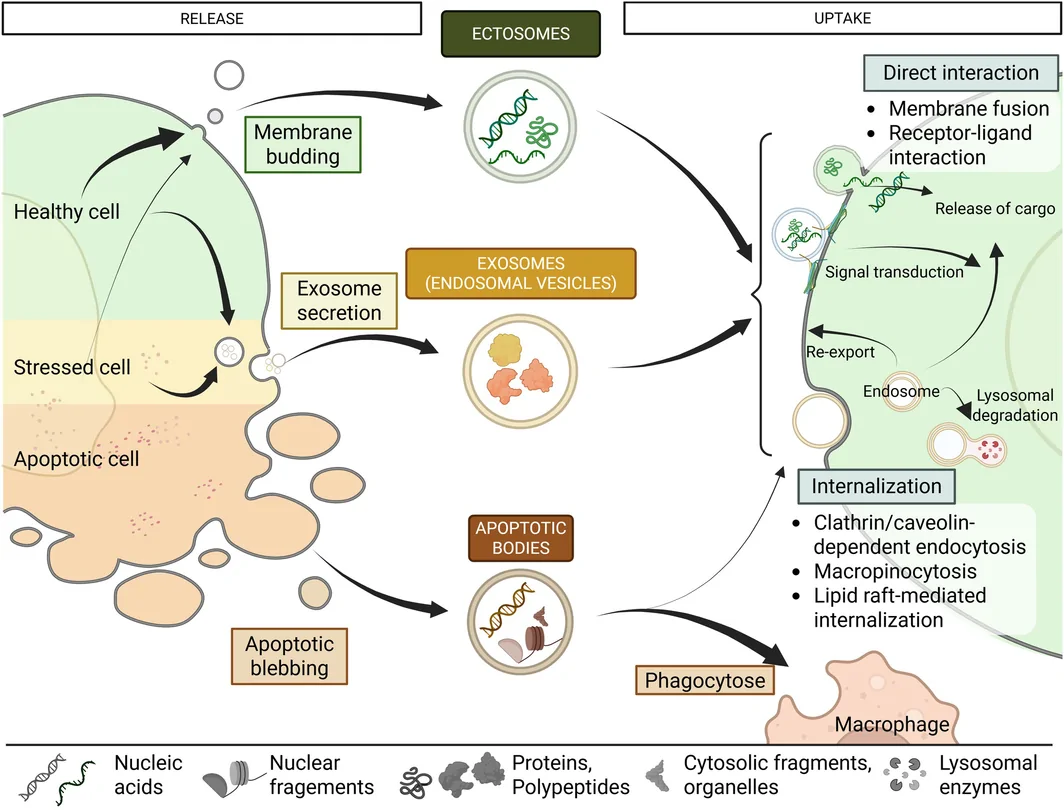
EV release and uptake mechanism. EVs are released from healthy, stressed, or apoptotic donor cells through different pathways, including direct membrane budding, exosome secretion from MVB of the endosomal system, and apoptotic blebbing during cell death. Depending on their mode of biogenesis, vesicles are classified as ectosomes, exosomes, or apoptotic bodies. EV‐mediated signaling can occur through receptor activation on the target cell surface or via vesicle uptake, membrane fusion, and subsequent cargo delivery. Following internalization, some EVs are recycled, re‐exported, or directed to degradation pathways. Non‐vesicular extracellular nanoparticles with unclear biogenesis or uptake mechanisms, such as exomeres, and other EV subtypes, such as exophers, were not included for clarity. Created in BioRender. https://BioRender.com/syfog9l.

EVs are highly recognized versatile mediators of intercellular communication (Figure 1). Despite their small size and limited cargo capacity, EVs collectively provide a vast interactive surface within biological fluids, with estimates suggesting that their cumulative membrane area can exceed that of other circulating blood components such as leukocytes. This extensive membrane interface implies that EV‐mediated communication is not governed solely by their luminal cargo but also by the molecular composition of the vesicular membrane itself, which participates directly in cell–cell recognition, signaling, and biodistribution (Jahnke and Staufer 2024). EVs transport a broad spectrum of biomolecules, including proteins such as receptors, adhesion molecules, signaling mediators, and enzymes; lipids enriched in cholesterol, sphingomyelin, and other membrane‐stabilizing components; and multiple classes of RNA, including microRNAs (miRNAs), transfer RNA fragments (tRNAs), messenger RNAs (mRNAs), and other non‐coding RNAs. Proteins and lipids typically constitute the majority of EV mass, whereas nucleic acids represent a smaller but functionally significant fraction capable of modulating gene expression in recipient cells (Kang et al. 2020; Jeppesen et al. 2019; Andaloussi et al. 2013; Valadi et al. 2007; Ratajczak et al. 2006).

Importantly, the molecular composition of EVs is not a passive reflection of the cytoplasmic content of their cells of origin. Instead, multiple studies have demonstrated selective cargo sorting during vesicle biogenesis, resulting in EV molecular profiles that differ markedly from those of the donor cell cytoplasm (Janas et al. 2015; Valadi et al. 2007; Ratajczak et al. 2006). For instance, EVs released by human embryonic stem cells are enriched in transcripts and proteins associated with pluripotency, including transcription factors, cytokines, and receptors involved in stem cell maintenance and self‐renewal, highlighting the capacity of EVs to transfer cell‐type specific regulatory signals (Ratajczak et al. 2006). EVs influence recipient cells through multiple modes of interaction, including ligand–receptor engagement at the cell surface, direct membrane fusion that releases vesicular cargo into the cytoplasm, and internalization through endocytic pathways such as clathrin‐dependent endocytosis, macropinocytosis, and phagocytosis. The efficiency and specificity of these interactions are strongly influenced by the molecular composition of the EV surface. In particular, the vesicular membrane is decorated with proteins, lipids, and adsorbed extracellular molecules that together form a dynamic “protein corona,” which modulates vesicle recognition, cellular uptake, and tissue distribution in vivo (Jeppesen et al. 2024). Consequently, both EV cargo and membrane architecture contribute to their function as complex signaling platforms that coordinate communication between cells within tissues and across systemic circulation.

### 1.1 Surface Composition, Dynamics, and Adaptability of EVs

Tetraspanins such as cluster of differentiation (CD) proteins CD9, CD63, and CD81 are abundant, canonical EV markers. While CD63 is occasionally mislabeled as LAMP3 in historical databases, true dendritic cell LAMP3 (DC‐LAMP/CD208) is a distinct lysosomal glycoprotein; thus, this review exclusively uses CD63 to avoid confusion (de Saint‐Vis et al. 1998; Salaun et al. 2004; Nazli et al. 2022). Functional knockouts in MCF7 cells demonstrate that these tetraspanins are not strictly mandatory for vesicle fusion or uptake (Tognoli et al. 2023). Instead, a compensatory redundancy exists within this network; for example, CD63 is frequently upregulated in the absence of CD9 (Fan et al. 2023). Live‐cell tracking in HeLa cells reveals that spatial residency dictates vesicle budding routes: CD63 is enriched in endosomes and marks endosome‐derived vesicles, whereas CD9 and CD81 localize to the plasma membrane to label small ectosomes. Co‐localization occurs primarily where these pathways intersect, such as at multivesicular bodies (MVBs) and the plasma membrane (Mathieu et al. 2021). Supporting this, deleting the endocytic motif of CD63 in HEK293 cells redirects it to the plasma membrane, increasing its co‐budding with CD9 fourfold, while adding an endocytic signal to CD9 targets it to endosomes to increase co‐budding with wild‐type CD63, confirming that local cellular residency drives cargo co‐loading (Fordjour et al. 2022).

Vesicle biogenesis and release are further coordinated by components of the endosomal sorting complex required for transport (ESCRT), including tumor susceptibility gene 101 (TSG101/ESCRT‐I), ALG‐2‐interacting protein X (ALIX), vacuolar protein sorting 4 (VPS4), and syntenin‐1, which sort cargo into intraluminal vesicles. These pathways cooperate with soluble N‐ethylmaleimide‐sensitive‐factor attachment protein receptor (SNARE) proteins to regulate MVB movement, maturation, and plasma membrane fusion (Jeppesen et al. 2023). Downstream binding and functional RNA transfer to recipient cells are mediated by surface integrin β1 and fibronectin, highlighting the importance of extracellular matrix interactions (Elsharkasy et al. 2025). Alternatively, receptor‐independent direct fusion can occur, driven by lipid composition and local pH. Cryo‐electron microscopy shows this proceeds via a viral‐like hemifusion mechanism (Morandi et al. 2022). Internalized vesicles frequently colocalize with low‐pH endosomal and lysosomal compartments; utilizing luciferase‐tagged cargo reporters combined with subcellular fractionation, studies demonstrate that approximately 20%–30% of internalized EVs successfully release their cargo into the recipient cytoplasm (Bonsergent et al. 2021). Importantly, this machinery is highly responsive to cellular stress. Genotoxic insults, oxidative stress, and chronic inflammatory cues directly engage p53‐dependent and NF‐κB‐dependent transcription pathways. This stress‐induced activation upregulates key regulatory Rab guanosine triphosphatases (GTPases), specifically Rab27a and Rab27b, which accelerate the transport, peripheral translocation, and docking of MVBs at the cell cortex. This network substantially enhances MVB fusion with the plasma membrane, shifting the overall rate of vesicle production and altering the secretory landscape. Ultimately, because EVs mirror the molecular and pathological states of their donor cells, understanding these interactive dynamics provides a critical conceptual framework for utilizing EVs as indicators of cellular dysfunction or disease across homeostatic and aging contexts (Phan and Reed 2024).

### 1.2 Methodological Considerations for EV Studies in Aging

Distinguishing EVs subtypes based on their biogenesis remains a central challenge. Exosomes and ectosomes overlap heavily in size and density, and lack universally specific biochemical markers, contributing to historical nomenclature inconsistencies in literature and commercial kits. Because no single isolation method provides complete purity, each physical separation mechanism introduces distinct contamination profiles that increase the risk of data misinterpretation (Table 1). To ensure standardization, this review follows Minimal Information for Studies of Extracellular Vesicles (MISEV) guidelines: unverified vesicles under 200 nm are termed small EVs (sEVs), and those over 200 nm are large EVs (Welsh et al. 2024).

**TABLE 1:** Technical framework and methodological constraints of EV isolation techniques.

Isolation method	Physical principle	Key contaminants and limitations	References
Differential ultracentrifugation and density gradients	Particle size and buoyant density	High risk of co‐sedimentation with soluble proteins, HDLs, and VLDLs.	Dong et al. (2020); Helwa et al. (2017)
Size exclusion chromatography and filtration	Hydrodynamic radius/molecular size	Cannot completely segregate sEVs from similarly sized chylomicrons and VLDLs.	Dong et al. (2020); Star et al. (2025)
Polymer‐based precipitation	Solubility and hydration layer alterations	High particle yield but very low purity; co‐precipitates non‐vesicular proteins and systemic RNA.	Dong et al. (2020); Star et al. (2025)
Affinity capture	Surface marker interactions	Biased toward specific tetraspanin‐positive sub‐populations; misses marker‐negative variations.	Star et al. (2025)

In aging cohorts, these methodological challenges are severely amplified. Age‐associated changes such as impaired endo/lysosomal clearance, altered membrane lipid dynamics, and senescence‐driven vesicle shedding drastically increase EV heterogeneity and shift subtype proportions. Concurrently, non‐vesicular background factors (lipoproteins, macromolecular protein aggregates, and inflammatory mediators) rise systemically, obscuring the distinction between true EVs and co‐isolated contaminants (Brennan et al. 2020; Dong et al. 2020; Noren Hooten 2020; Tian et al. 2020). Furthermore, age‐related physiological shifts alter downstream vesicle dynamics and clearance, differently across preclinical models and human clinical cohorts. In animal models, tracking these altered dynamics reveals direct links to tissue‐level senescence. Studies of EV‐associated miR‐29 in female mice indicate age‐linked shifts in small RNA cargo that correlate with altered downstream immune signaling, supporting the hypothesis that altered uptake kinetics and remodeled cargo drive immune senescence (Kern et al. 2023). Crucially, investigators emphasize that strict caution with isolation methodologies is critical, as co‐isolated background elements can easily skew structural and functional interpretations of age‐related small RNA payloads.

Paralleling these preclinical findings, human physiological datasets confirm that aging modifies vesicle handling and the systemic background environment, though direct mechanistic translation remains challenging. Human Peripheral Blood Mononuclear Cell (PBMC) assays demonstrate that EVs from older donors undergo accelerated, dose‐ and time‐dependent internalization by primary monocytes and B cells in vitro. This selective uptake alters recipient activation states, upregulating MHC‐II/CD80 on monocytes and CD25/CD80 on B cells. While it is a compelling working hypothesis that heightened B‐cell consumption may account for the lower overall circulating free EV levels observed with age, senescent secretory changes or altered systemic clearance kinetics cannot be ruled out as primary drivers (Eitan et al. 2017). These vesicular shifts occur against a background of subclinical systemic inflammation; for instance, asymptomatic older adults display a tighter clinical coupling between C‐reactive protein (CRP) and erythrocyte sedimentation rate (ESR), alongside an increased prevalence of rheumatoid factor without overt autoimmune disease, directly impacting baseline systemic EV heterogeneity (Suliman 2026).

Ultimately, an exact understanding of isolation performance remains key. While age‐induced environmental shifts complicate vesicle tracking, implementing objective and target‐quantified benchmarking protocols creates a distinct clinical opportunity to identify reproducible, validated biomarkers for age‐related diseases and functional decline, rather than relying on unstandardized isolation pools (Noren Hooten 2020; Raez‐Meseguer et al. 2026a, 2026b).

### 1.3 EVs in Normal Hematopoiesis

HSPCs internalize niche vesicles efficiently. Murine hematopoietic stem cells (HSCs) take up PKH67‐labeled EVs from the BM stromal cell line MS‐5 within 4 h. Co‐culture models show this uptake drives transcriptional alterations in recipient HSPCs, modulating apoptosis and proliferation pathways (Batsali et al. 2020; Preciado et al. 2019). Mechanistically, the TGF‐β/SMAD pathway serves as a primary axis of this communication. In vitro blockade using an ALK5 inhibitor (TGF‐β receptor 1 inhibitor) in MS‐5 cells increases total EV shedding, but these vesicles are severely depleted of p‐SMAD2, which correlates with impaired HSC maintenance and loss of quiescence (Gautheron et al. 2023). Beyond stroma, osteoblast‐derived EVs directly instruct progenitor fates. Proliferation assays and in silico predictions show osteoblastic EVs alter target cell cycle progression and proliferation gene networks (Morhayim et al. 2016). These vesicles carry stress‐induced tRNA fragments such as 5′‐ti‐Pro‐CGG‐1 which promote granulocytic and monocytic differentiation in vitro while reinforcing cell‐mediated antimicrobial functions (Kfoury et al. 2021).

These findings are validated via preclinical mouse models in vivo. Systemic inhibition of EV release via calpeptin in C57BL/6J mice reduces BM cavity EV abundance and depletes SMAD2 and pSMAD2 inside remaining intra‐marrow vesicles without altering size distribution (Gautheron et al. 2023). This supports the hypothesis that steady‐state SMAD signaling relies dynamically on active vesicle secretion. Furthermore, osteoblast‐specific GFP reporter mice demonstrate that osteolineage‐derived EVs are preferentially internalized in vivo by granulocyte–macrophage progenitors (GMPs). This targeted transfer augments myeloid progenitor expansion and colony‐forming capacity. Expanding the parental osteoblast pool via intermittent parathyroid hormone (PTH) therapy or constitutive PTH receptor activation increases functional EV transfer to GMPs, accelerating myeloid recovery following irradiation stress and improving survival against *Candida albicans* challenges (Kfoury et al. 2021).

Conversely, human evidence regarding steady‐state, homeostatic EV‐mediated hematopoiesis remains severely limited, complicating direct translation. While MSC‐EVs carry angiogenic proteins that promote endothelial remodeling and immunomodulation, their precise baseline contributions to human HSC regulation are unmapped (Batsali et al. 2020). This gap reflects technical hurdles, such as isolating cell‐type specific EVs from intact human bone marrow aspirations in vivo. Additionally, most human datasets prioritize disease‐altered or malignant EV communication over steady‐state physiology. Consequently, how major human bone marrow niche populations, including chimeric antigen receptor (CAR) cells, Lepr+ stromal cells, Schwann cells, megakaryocytes, and marrow adipocytes orchestrate EV biogenesis, cargo loading, and downstream signaling to regulate human HSC maintenance during steady‐state hematopoiesis remains an important yet underexplored area of investigation.

## 2 EVs as Regulators of Hematologic Aging

### 2.1 EVs in Aging: Mechanisms, Remodeling, and Therapeutic Potential

Chronological aging drives a progressive accumulation of senescent cells across tissues, though whether this reflects increased induction, impaired immune clearance, or both remains incompletely understood (Ovadya et al. 2018; Suryadevara et al. 2024; Wang et al. 2022). Within the BM microenvironment, senescent MSCs and niche populations shape tissue aging by delivering the senescence‐associated secretory phenotype (SASP), where EVs act as stable mediators of long‐range intercellular signaling (Wang, Han, et al. 2024). Rather than acting as passive bystanders, senescent cells utilize altered EV networks to create a pro‐inflammatory, pro‐survival microenvironment that disrupts normal hematopoiesis and favors the competitive expansion, migration, and therapy resistance of mutated clones harboring oncogenic mutations (Salazar‐Terreros and Vernot 2022).

In vitro, murine models demonstrate that senescent cells use EVs to propagate aging phenotypes. Senescent endothelial cells exhibit an approximate 3‐fold increase in sEV shedding. These vesicles are selectively enriched with miR‐21‐5p and miR‐217; when internalized by healthy endothelial cells, this miRNA cargo downregulates sirtuin 1 and DNA methyltransferase 1, reducing proliferation and inducing secondary senescence (Mensa et al. 2020). Similarly, aging enriches the miR‐183 cluster (miR‐96/‐182/‐183) in bone‐derived EVs, where vesicular miR‐183‐5p suppresses healthy BM‐MSC proliferation and osteogenesis (Davis et al. 2017). Furthermore, EVs from aged mouse skeletal muscle carry increased miR‐34a, miR‐21‐5p, miR‐217, and SASP proteins, spreading senescence to recipient BM stem and endothelial cells (Fulzele et al. 2019; Mensa et al. 2020).

Conversely, youthful murine cells secrete protective vesicles. Tropomyosin‐1 (TPM1) is a key mechano‐regulatory cargo packaged into young osteocyte‐derived EVs that reinforces actin cytoskeletal organization in target BM‐MSCs, supporting osteogenesis (Brielle et al. 2021; Wang, Lin, et al. 2024). With age, senescent osteocytes deplete vesicular TPM1; treating BM‐MSCs with these aged vesicles diminishes cytoskeletal organization, upregulates the master adipogenic regulator peroxisome proliferator‐activated receptor gamma (*Pparg*), and skews BM‐MSC fate from bone formation toward adipogenesis (Wang, Lin, et al. 2024). Paralleling this, genetic modeling in human induced pluripotent stem cells (iPSCs) shows that a complete loss of TPM1 significantly enhances early hematopoietic progenitor cell (HPC) formation in vitro and in vivo by augmenting tumor necrosis factor alpha (TNF‐α) signaling (Wilken et al. 2024). In contrast, EVs from youthful human iPSCs and MSCs are enriched in antioxidant enzymes, including Peroxiredoxins (PRDX) 1 and 2, which successfully attenuate oxidative stress and partially rejuvenate aged HSCs (Kulkarni et al. 2018; Liu et al. 2019).

Preclinical mouse and rat models confirm that aged immune cells secrete deleterious vesicles that reshape systemic health and the marrow cavity. EVs derived from the BM monocytes and macrophages of aged mice (20–24 months) induce systemic aging phenotypes when transplanted into young recipients, causing increased senescence burdens across multiple tissues, impaired glucolipid metabolism, reduced insulin sensitivity, and accelerated bone loss (Hou et al. 2024). Locally, grancalcin (GCA) expression is increased 10–15 fold in neutrophils and M1‐like macrophages from aged rats (Li et al. 2021). Systemic administration of recombinant GCA (rGCA) in mice reduces osteocalcin+ osteoblasts and TRAP+ osteoclasts while enhancing marrow adiposity, remodeling the BM niche into a low‐turnover state unfavorable for healthy hematopoiesis (Li et al. 2021). Immunologically, aging is also associated with a systemic decline in beneficial immune‐derived CD9^+^ and CD31^+^ EV subpopulations and their mitochondrial cargo, indicating impaired metabolic support within the aging niche (Zhang et al. 2020).

In human clinical cohorts, chronological aging drives a steady accumulation of senescent MSCs and niche‐resident cell types within the BM cavity. These senescent human MSCs exhibit profound signaling alterations that disrupt normal hematopoietic regulation, compromising niche integrity and activating pro‐survival pathways to support malignant cell survival, migration, and therapy resistance in hematologic malignancies (Salazar‐Terreros and Vernot 2022). Ultimately, the aging EV landscape reflects a homeostatic imbalance characterized by an increased release of pro‐senescent EVs from damaged cells and a concomitant decline in protective EVs from youthful stromal and immune populations. Managing these alterations creates a distinct clinical opportunity to identify reproducible biomarkers for age‐related functional decline (Salazar‐Terreros and Vernot 2022).

### 2.2 Changes in EV Abundance and Biogenesis With Age

**FIGURE 2:**
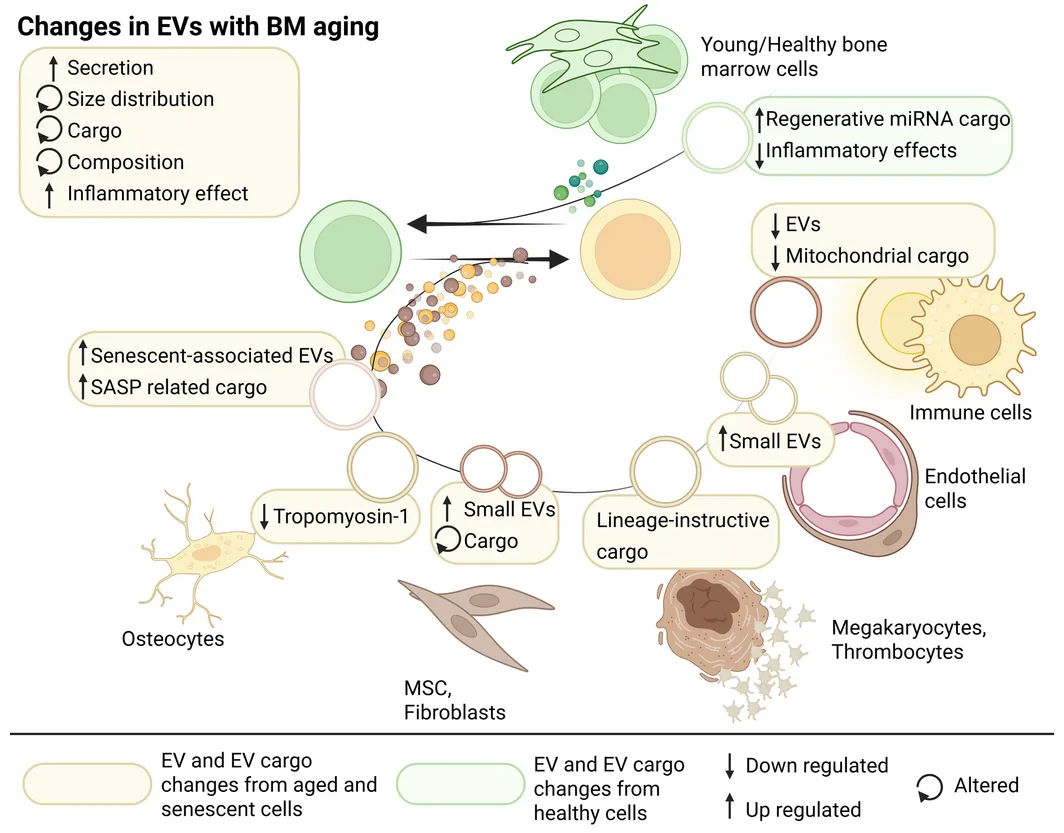
Alterations of EVs in the BM during aging. Aging affects EV production, cargo composition, and intercellular communication in multiple BM cell types, displayed by upward, downward, and circular arrows. Senescent or aged EVs can induce aging‐related features in recipient cells, whereas juvenile EVs have rejuvenating effects, promoting more youthful cellular phenotypes. Created in BioRender. https://BioRender.com/f1lsvvz.

Aging alters EV abundance and biogenesis, driven by cellular stress and damage‐response pathways. At the molecular level, DNA damage, oxidative stress, and inflammatory cues associated with senescence activate p53‐ and NF‐κB (Jahnke and Staufer 2024) dependent transcriptional programs that enhance endosomal trafficking, MVB formation, and vesicle release (Feng 2010; Lespagnol et al. 2008; Tesei et al. 2021; Yu et al. 2006; Yu et al. 2009). These pathways increase the engagement of ESCRT machinery and Rab GTPases, particularly Rab27a and Rab27b, which regulate MVB docking and fusion with the plasma membrane. Through this coupling of genotoxic stress and inflammatory signaling, senescent cells generate an EV‐rich secretory landscape that alters circulating concentrations, size distribution, and composition locally and systemically (Yin et al. 2021) (Figure 2).

In vitro models show that murine cells directly link genotoxic stress to altered EV output. Senescence‐linked activation of p53 and NF‐κB under oxidative or inflammatory stress drives the transcription of machinery that accelerates MVB biogenesis and vesicle shedding (Feng 2010; Yu et al. 2009; Lespagnol et al. 2008; Yu et al. 2006). Within simulated BM niche environments, aged and senescent murine stromal cells exhibit cell‐autonomous changes in EV biogenesis, producing vesicles with altered protein and microRNA cargo that disrupt normal stem cell homeostasis (Chen et al. 2019; Goloviznina et al. 2016; Gong et al. 2020; Hu et al. 2020; Kulkarni et al. 2018; Mas‐Bargues et al. 2020; Tian et al. 2020). Corresponding human in vitro models demonstrate that senescent human cell types modify their secretory profile through identical ESCRT‐ and Rab‐GTPase‐dependent pathways (Tesei et al. 2021). Chronic stress upregulates human Rab27a/b expression, increasing MVB plasma membrane docking and vesicle release. Functional assays show that this age‐dependent cargo enrichment including inflammatory mediators, mitochondrial fragments, and senescence‐associated microRNAs directly impairs downstream cellular communication.

Preclinical rodent studies reveal highly variable, species‐specific shifts in circulating EV metrics across the lifespan. In murine models, plasma EVs from aged mice (18–21 months) display reduced mean vesicle size but paradoxically higher total particle concentrations and an enrichment of canonical markers (CD63, CD81, TSG101), indicating qualitative remodeling rather than simple depletion (Alibhai et al. 2020). Conversely, rat serum across age groups (3 vs. 21–23 months) reveals that total EV concentration significantly decreases with advancing age (Zhang et al. 2021). Within the local BM niche of aged rodents, increased EV release from senescent stromal, osteolineage, and immune cells collectively reshape the hematopoietic microenvironment. These niche‐derived EVs deliver an altered cargo landscape to HSPCs, driving canonical features of hematopoietic aging, including diminished self‐renewal, myeloid‐biased differentiation, and impaired regenerative capacity (Chen et al. 2019; Goloviznina et al. 2016; Gong et al. 2020; Hu et al. 2020; Kulkarni et al. 2018; Mas‐Bargues et al. 2020; Tian et al. 2020).

In clinical cohorts, the consensus regarding age‐associated shifts in plasma EV concentration is highly varied, reflecting complex systemic dynamics across human life stages. Cross‐sectional tracking has shown that EV concentration decreases significantly with age in human plasma when comparing younger cohorts to middle‐aged cohorts (30–60 years old) (Eitan et al. 2017). Conversely, other clinical studies have observed a significantly larger number of circulating EVs in bodily fluids from elderly subjects compared to young controls, namely when comparing plasma from individuals aged 20–30 to those around 80 years old (Alique et al. 2017; Borghesan et al. 2019). Beyond absolute abundance, human aging impairs the systemic functional activity of circulating vesicle populations. Plasma EV‐like particles isolated from older individuals exhibit reduced internalization by leukocytes and diminished functional activity in endothelial and immune assays, suggesting compromised EV uptake and signaling within hematopoietic compartments (Alibhai et al. 2020; Eitan et al. 2017). This clinical defect correlates with the accumulation of deleterious cargo, which exacerbates chronic inflammation, immune decline, and susceptibility to age‐associated hematologic diseases.

### 2.3 Remodeling of EV Cargo During Aging

Aging extensively remodels EV cargo, reflecting fundamental shifts in cellular metabolism, inflammatory tone, and epigenetic regulation. Within the hematopoietic system, these altered vesicle compositions propagate senescence‐associated signals through the BM microenvironment. Rather than acting as passive biomarkers, aged EVs function as active drivers of niche remodeling and HSPC dysfunction by carrying altered miRNA and protein payloads.

In vitro models show that age‐associated miRNA remodeling within the local murine skeleton directly compromises stromal support. EVs isolated from the BM interstitial fluid (supernatant) of aged mice (24–28 months) are enriched in the miR‐183 cluster (miR‐96, miR‐182, and miR‐183). When primary murine bone marrow stromal cells (BMSCs) internalize these aged vesicles, proliferation is suppressed, osteogenic differentiation is inhibited, and cellular senescence is induced. Mechanistically, miR‐183‐5p alone drives this stromal decline by downregulating heme oxygenase‐1 (Hmox1), a cytoprotective enzyme required for redox homeostasis (Davis et al. 2017). This local deterioration is exacerbated by systemic sources: EVs from aged murine skeletal muscle are enriched in miR‐34a and directly induce secondary senescence and loss of viability in BM‐MSCs ex vivo.

In human cellular systems, age‐dependent cargo variations translate into distinct functional impairments during stromal‐to‐hematopoietic communication. MSCs from older donors produce EVs with significantly altered miRNA profiles. In vitro exposure of human CD34^+^ HSPCs to these aged MSC‐derived EVs dynamically alters HSPC viability, cell cycle gene expression, and clonogenic colony output, showing that aged stromal vesicles fail to deliver proper homeostatic support. Similarly, treating human HSPCs directly with aged circulating plasma EVs triggers explicit features of cellular senescence, marked by a diminished proliferative capacity and skewed differentiation potential (Grenier‐Pleau et al. 2020).

Preclinical mouse models validate that age‐altered EV cargo acts as a functional messenger connecting peripheral tissues to the BM cavity. Localized elevation of the miR‐183 cluster within aged mouse bone fluids coordinates a targeted microenvironmental shift, disrupting healthy bone remodeling and niche integrity in vivo (Davis et al. 2017). Concurrently, elevated transport of miR‐34a via muscle‐derived EVs demonstrates that aging‐related cargo remodeling operates cross‐tissue, enabling peripheral organs to infiltrate the BM compartment and accelerate microenvironmental aging.

Observational studies of circulating human EV profiles confirm that aging drives highly reproducible shifts in the systemic miRNA landscape across the lifespan. Plasma‐derived EVs from elderly clinical donors display distinct, age‐specific miRNA signatures compared to young individuals (Grenier‐Pleau et al. 2020). Specifically, circulating plasma EVs from elderly subjects are enriched for miR‐29 and miR‐96, but depleted of miR‐146. These specific cargo shifts correlate with the dysregulated expression of central cell cycle regulators such as cyclin‐dependent kinase (CDK) 6 and CDKN1A (p21) in recipient target cells, supporting a chronic, senescence‐associated transcriptional program within the aging human hematopoietic system (Abbasi Sourki et al. 2023) (Table 2).

**TABLE 2:** Summary of EV cargo remodeling and functional impacts in aging.

Species/System	EV source compartment	Shifted cargo component	Downstream cellular target	Primary phenotypic and functional outcome	References/Source
Murine	BM Interstitial Fluid	↑ miR‐183 cluster (miR‐96/−182/−183)	Primary BMSCs	Suppresses proliferation, blocks osteogenesis, and induces senescence via Hmox1 downregulation.	Davis et al. (2017)
Murine	Skeletal Muscle	↑ miR‐34a	Bone Marrow MSCs	Drives cross‐tissue senescence propagation and reduces stromal cell viability ex vivo.	Fulzele et al. (2019)
Human Systems	Bone Marrow MSCs	Altered homeostatic miRNA profile	CD34+ HSPCs	Distorts cell cycle gene expression networks, alters viability, and reduces clonogenic output.	Fichtel et al. (2022)
Human Systems	Circulating Plasma	Elderly miRNA signature	Human HSPCs	Instigates cellular senescence, restricts expansion, and disrupts normal differentiation.	Grenier‐Pleau et al. (2020)
Human/Clinical	Circulating Plasma	↑ miR‐29, ↑ miR‐96; ↓ miR‐146	Hematopoietic System	Modulates CDK6 and p21 expression; executes systemic senescence‐associated transcription programs.	Abbasi Sourki et al. (2023)

### 2.4 EVs, Telomere Attrition, and Inflammaging in the Aging Hematopoietic System

Telomere attrition directly regulates EV biology, providing a mechanistic link between genomic instability, chronic inflammation, and tissue decline. Systemic low‐grade inflammaging and genotoxic stress reshape the EV landscape, transforming these particles from homeostatic mediators into primary conveyors of chronic inflammatory signals. Within the BM niche, this dysregulated EV network intersects with cell‐intrinsic HSC stress, disrupting BMSC fitness, altering lineage choice, and driving the functional decline characteristic of the aging hematopoietic system.

Models from in vitro studies show that age‐associated inflammatory environments and genetic stressors fundamentally modify vesicle signaling and recipient response pathways. EVs released by senescent murine macrophages are highly enriched in pro‐inflammatory microRNAs, specifically miR‐21a‐5p and miR‐155‐5p, which drive active secretion of Interleukin1 beta (IL‐1β) and IL‐6 in healthy recipient macrophages via direct activation of the SIRT1/NF‐κB axis (Xiao et al. 2022). Concurrently, EVs from aged murine MSCs display altered miRNA profiles and reduced macrophage internalization efficiency, reinforcing inflammatory feedback loops (Huang et al. 2019). Within the local marrow cavity, exposure to aged, miR‐183‐5p‐enriched vesicles directly suppresses primary BMSC proliferation and downregulates osteogenesis (Davis et al. 2017). Furthermore, pharmacologic or inflammatory stressors like granulocyte‐colony stimulating factor (G‐CSF) actively remodel this intra‐marrow EV landscape by promoting the accumulation of miR‐126–containing EVs; when internalized by HSPCs, stromal cells, and endothelial cells, this vesicular cargo suppresses surface expression of VCAM‐1, weakening α4β1 integrin–mediated adhesion to facilitate cell mobilization (Davis et al. 2017; Jiang et al. 2017). In human systems, embryonic and adult developmental stages dictate the performance of stromal vesicle cargo, where adult human BMSC‐derived EVs support CD34^+^ HSPC expansion far better than fetal equivalents due to an enrichment of mitochondrial ATP synthesis, protein‐folding, and redox proteins, alongside the miR‐99b/let‐7e/miR‐125a cluster (Ghebes et al. 2021). However, this supportive capacity is lost when human cells undergo telomere attrition; these stressed cells enrich their secreted vesicle membranes with inflammatory and lipid‐metabolism‐associated proteins, shifting their role from baseline maintenance to the active induction of recipient cellular stress.

Preclinical rodent models validate that telomere erosion and microenvironmental remodeling explicitly alter EV abundance, payload, and tissue‐level interactions in vivo. In telomerase reverse transcriptase‐deficient Tert^−/−^ mouse models, plasma EVs undergo severe quantitative and qualitative remodeling matching the progression of telomere erosion. Specifically, third generation (G3) Tert^−/−^ mice demonstrate an approximate 36% reduction in circulating plasma EV abundance compared with wild‐type controls, and these vesicles progressively lose physical telomeric DNA cargo (Gong et al. 2025). Functionally, EVs isolated from these G3 Tert^−/−^ mice act as toxic vectors, rapidly inducing pro‐inflammatory cytokines (including IL‐6) in BM‐derived macrophages and compromising primary cortical neuron viability in vivo (Gong et al. 2025). At the stem cell level, single‐cell analyses of telomerase‐deficient mice reveal that HSCs with critically short telomeres suffer profound cell‐intrinsic changes, existing in a state of chronic metabolic activation and sustained upregulation of innate immune and interferon (IFN) signaling programs (Thongon et al. 2021). Rather than triggering apoptosis, this continuous IFN axis acts as a functional constraint, biasing stem cells toward megakaryocytic differentiation, elevating metabolic demand, and diminishing long‐term self‐renewal to drive stem cell exhaustion (Thongon et al. 2021). This lineage‐biasing mechanism mirrors the activity of diverse vesicle subsets in the steady‐state marrow cavity, where megakaryocyte‐derived EVs directly instruct healthy HSPCs toward megakaryocytic differentiation through the delivery of lineage‐specific cargo, tying platelet output directly to intercellular vesicle exchange (Goloviznina et al. 2016; Jiang et al. 2017; Kumar et al. 2018).

Importantly, interventional studies show that this pro‐inflammatory signaling can be reversed; systemic administration of EVs purified from young donor mouse serum into aged mice dramatically reduces circulating levels of IL‐6, IL‐1β, and TNF‐α, while partially rejuvenating thymic architecture and general immune function (Wang et al. 2018). Similarly, treating the niche with preconditioned MSC‐derived EVs counteracts local inflammaging by suppressing pro‐inflammatory macrophage activation and restoring baseline metabolic homeostasis within the BM cavity (Gorgun et al. 2022).

Observational studies of human aging cohorts validate that genomic instability and systemic inflammation track closely with remodeled circulating vesicle profiles across the human lifespan. Clinical datasets demonstrate that plasma EVs from aged individuals exhibit a progressive loss of telomeric DNA cargo, matching the systemic telomere attrition observed in peripheral blood cells (Gong et al. 2025). Concurrently, age‐related modifications in human plasma EV cytokine payloads and immune‐cell‐associated surface markers correlate directly with clinical measures of immunosenescence and altered lymphoid regulation (Zhang et al. 2024). This altered systemic EV pool feeds back into the human BM compartment via chronic low‐grade inflammatory cues, further eroding baseline hematopoietic function (Goldberg 2021; Zhang et al. 2024). Over time, this chronic inflammatory state and the accumulation of niche‐derived SASP vesicles provide a critical clinical bridge linking telomere erosion and skewed lineage output to the development of age‐associated clonal hematopoiesis and progressive hematologic disease (Goldberg 2021).

### 2.5 Rejuvenation Potential of EVs in the Aging BM Niche

sEVs can extend lifespan and restore youthful physiological function in aged organisms by acting as vehicles for regulatory microRNAs, metabolic enzymes, and replication factors that reprogram senescent cells toward a regenerative state. By restoring mitochondrial function, dampening senescence pathways, preserving telomere integrity, and reinforcing stem cell support within the BM niche, youthful EVs present a promising therapeutic avenue to counter hematopoietic aging, restore niche function, and reduce vulnerability to age‐associated hematologic and skeletal diseases.

**FIGURE 3:**
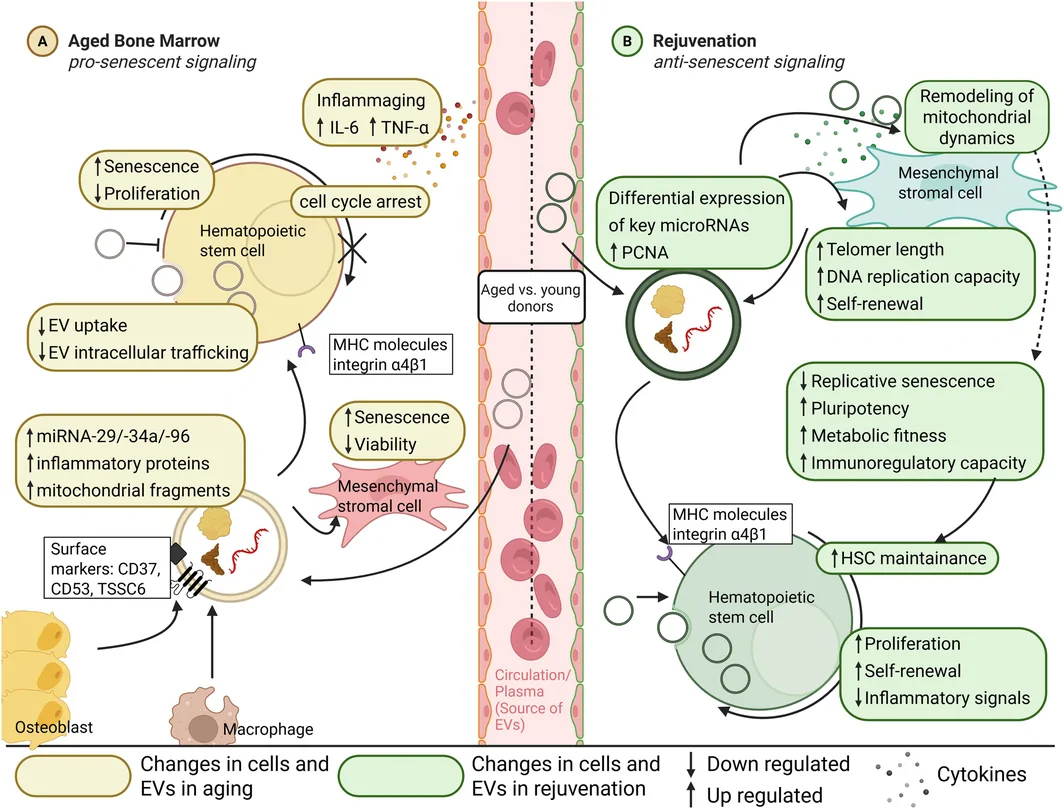
EVs define aging and rejuvenation dynamics in the BM niche. The left panel illustrates an aged niche with heightened inflammatory tone, altered EV communication, and reduced hematopoietic and stromal performance. The right panel illustrates EV‐based rejuvenation, in which youthful or engineered vesicles convey restorative signals that re‐establish supportive niche interactions and balanced hematopoiesis. Created in BioRender. https://BioRender.com/9svchkk.

In vitro models demonstrate that neonatal components can directly repair age‐related bioenergetic decline in BMSCs. EVs derived from juvenile mice and engineered to activate mitophagy via Tomm7‐mediated recruitment of the Pink1/Parkin axis effectively restore mitochondrial quality control in aged BMSCs, eliminating dysfunctional mitochondria to improve cellular bioenergetics and counteract conditions driving skeletal and hematopoietic decline (Zheng et al. 2025). Parallel evidence in human cellular systems demonstrates that youthful EVs derived from stem cell and plasma compartments directly rescue both HSC and stromal fitness. For instance, EVs isolated from young human plasma significantly enhance the proliferation and self‐renewal of umbilical cord blood–derived HSCs in vitro, whereas EVs from aged donors induce explicit senescence features and promote myeloid‐biased differentiation (Abbasi Sourki et al. 2023) (Figure 3). Additionally, human sEVs isolated from the secretomes of stem cells from human exfoliated deciduous teeth alleviate replicative senescence in human BMSCs by remodeling mitochondrial dynamics through the regulation of dynamin‐related protein 1 (Drp1) translocation, preserving the metabolic fitness and immunoregulatory capacity of the niche (Peng et al. 2024). Youthful human vesicles from neonatal sources also successfully deliver proliferating cell nuclear antigen (PCNA) into aged human BMSCs, increasing telomere length, DNA replication capacity, and self‐renewal (Lei et al. 2021).

Preclinical mouse models confirm that the systemic or targeted administration of youthful vesicle populations reverses multi‐organ aging phenotypes in vivo. Systemic administration of young plasma sEVs into aged mice rapidly reduces canonical senescence markers (including SA‐ β‐gal, p21, and p16), lowers reactive oxygen species (ROS) accumulation, restores cellular proliferative capacity, and rescues mitochondrial function across multiple organ systems. Mechanistically, this systemic rejuvenation is driven by the delivery of specific microRNAs including miR‐144‐3p, miR‐149‐5p, and miR‐455‐3p which upregulate Peroxisome proliferator‐activated receptor gamma coactivator 1 alpha (PGC‐1α) to actively support mitochondrial biogenesis (Chen et al. 2024). These interventional and functional transfer studies demonstrate that the EV‐driven delivery of PCNA into aged marrow compartments translates into improved in vivo regenerative outcomes, specifically accelerating bone formation and tissue repair processes tightly linked to BM progenitor function (Lei et al. 2021). Furthermore, the in vivo deployment of engineered juvenile murine EVs targeting the Pink1/Parkin mitophagy axis successfully counteracts age‐related skeletal degeneration, directly improving the microenvironmental cues required for effective HSC maintenance by rescuing intrinsic stromal function (Zheng et al. 2025).

Observational and correlative clinical data establish that circulating EVs undergo predictable changes across the human lifespan, acting as systemic regulators of hematopoietic aging. Human circulating EVs exhibit divergent, age‐dependent cargo profiles when comparing young and elderly populations (Figure 3). The opposing biological effects observed between young and aged plasma vesicle tracking correlate clinically with the differential expression of key microRNAs, including miR‐29, miR‐96, and miR‐146, which regulate cell cycle control, chronic inflammatory signaling, and stemness pathways in downstream human target organs (Abbasi Sourki et al. 2023). Ultimately, the age‐associated loss of these protective, youthful EV signatures directly correlates with a compromised human BM microenvironment, driving the onset of myeloid lineage bias, bone attrition, and a heightened clinical vulnerability to age‐related hematologic diseases (Abbasi Sourki et al. 2023).

### 2.6 EVs as Potential Stress Response Biomarkers in Hematologic Aging

EVs serve as sensitive indicators of cellular stress, reflecting the physiological and pathological state of their parental cells. In hematologic aging, cumulative stressors including chronic inflammation, oxidative damage, replicative exhaustion, and niche dysfunction progressively remodel EV composition, providing a dynamic, systems‐level snapshot of hematopoietic stress exposure and adaptive capacity. In vitro and ex vivo tracking models reveal that replicative stress induces explicit molecular shifts in murine EV cargo, loading vesicles with senescence‐associated signaling molecules, while age‐dependent alterations impair recipient cell uptake and intracellular trafficking pathways within the hematopoietic microenvironment (Alibhai et al. 2020). Parallel human systems confirm that age‐associated EV cargo modifications encode functional information about hematopoietic stress states well before overt clinical dysfunction manifests. At the molecular level, human vesicles enriched for stress‐ and senescence‐associated cargo, including inflammatory proteins, mitochondrial fragments, and regulatory microRNAs such as miR‐29, miR‐34a, and miR‐96, restrict proliferative capacity of human HSPCs and activate senescence transcriptional programs (Abbasi Sourki et al. 2023). In tandem, human cellular aging is characterized by impaired EV internalization, which directly alters downstream signaling kinetics (Eitan et al. 2017).

Preclinical mouse models provide functional evidence that systemic EVs reflect and dynamically modulate age‐associated inflammatory stress. In functional transfer experiments, EVs isolated from the serum of young mice (1–3 months) significantly attenuate chronic inflammation when administered systemically to 18‐month‐old aged mice, as demonstrated by a marked reduction in circulating IL‐6 levels and the partial restoration of T‐cell immune tolerance (Wang et al. 2018). These anti‐inflammatory effects extend to the central nervous system, occurring either through direct EV transit across the blood–brain barrier or via the suppression of peripheral inflammaging that secondarily influences neuroinflammation (Wang et al. 2018). While the precise molecular mediators within the cargo remain to be fully delineated, this interventional work establishes that EV cargo carries stress‐responsive, anti‐inflammatory signals that decline with age, highlighting circulating vesicles as functional readouts of systemic immune stress rather than passive byproducts of aging (Wang et al. 2018).

Observational studies of human EV profiles across the lifespan reveal that age‐dependent remodeling of circulating EV cargo occurs in otherwise healthy individuals and directly influences primitive hematopoietic behavior. Grenier‐Pleau and colleagues demonstrated that while absolute plasma EV concentration and vesicle size remain relatively stable across adulthood, the specific protein composition of these vesicles changes markedly with age (Grenier‐Pleau et al. 2020). In clinical correlation assays, EVs derived from middle‐aged and older human donors (greater than 40 years) significantly increased the colony‐forming capacity of umbilical cord blood‐derived CD34^+^ HSPCs compared with EVs isolated from younger individuals, notably without altering downstream lineage output (Grenier‐Pleau et al. 2020). This selective increase in colony number without lineage skewing indicates a targeted stimulation of primitive progenitors, consistent with a compensatory hematopoietic activation in response to chronic, low‐grade systemic stress (Grenier‐Pleau et al. 2020). Longitudinal profiling of this stress‐responsive EV cargo represents a promising clinical avenue to enable the early detection of hematopoietic dysfunction, distinguish between healthy and maladaptive aging trajectories, and monitor real‐time patient responses to anti‐inflammaging interventions.

### 2.7 EVs and Targeting of Distinct Cell Subsets in Hematopoietic Contexts

EV internalization is governed by vesicle surface proteins, lipids, and recipient cell receptors rather than random uptake. While EVs do not actively “home” via chemotaxis, selective target‐cell retention occurs, which is crucial during hematologic aging as stress‐responsive vesicles alter specific BM microenvironmental nodes (He et al. 2022). In vitro murine studies show that vesicle exchange is tightly regulated by inherited surface profiles, including tetraspanins (CD9, CD63, CD81, CD82, and CD151) and integrins. Notably, CD9 physically associates with c‐kit (CD117), a receptor highly expressed on HSPCs facilitating preferential vesicle recognition and internalization by primitive progenitors (Anzai et al. 2002). Human hematopoietic cells process these signals via diverse pathways, including lipid raft‐mediated endocytosis, macropinocytosis, phagocytosis, and direct membrane fusion. Human megakaryocyte‐derived EVs exploit lipid raft‐dependent endocytosis, macropinocytosis, and fusion to selectively enter HPCs (Butler et al. 2018), reinforcing the concept of “exosome fingerprinting” where shared surface signatures drive preferential internalization by homologous cells (Sancho‐Albero et al. 2019). Crucially, human models reveal that vesicle uptake does not inherently equate to functional influence, as internalized EVs are frequently routed to degradation or recycling pathways without altering recipient cell behavior (Muhandiram and Fazeli 2026).

Preclinical rodent models reveal a substantial gap between localized in vitro docking affinity and true systemic distribution, as comprehensive in vivo mapping of natural EV targeting specificity within the aging hematopoietic system remains limited. Most existing murine studies focus heavily on macro‐level EV biodistribution across major clearance organs (liver, spleen, and lungs) or on engineered therapeutic vehicles, rather than defining endogenous, cell‐subset‐specific vesicle trafficking inside the aging BM cavity (Liu and Wang 2023). In murine hematologic aging, it remains entirely unmapped how age‐associated changes in EV surface chemistry alter cell subset targeting in vivo, whether senescent‐derived vesicles preferentially target specific hematopoietic fractions versus supporting stromal cells, or how alternative endocytic mechanisms fluctuate across aged versus young murine tissues.

Similarly, human clinical data confirm that while EV cargo remodels with age to influence hematopoiesis, the precise surface receptor dynamics governing these interactions across human cell subsets remain poorly characterized. Current clinical evidence emphasizes that many systemic interactions are highly nonspecific and largely dictated by the surrounding tissue microenvironment and baseline endocytic capacity (Liu and Wang 2023). Furthermore, while human hematopoietic aging alters the surface receptor landscape of immune and bone marrow cells, how this receptor drift affects natural EV uptake fidelity has not been resolved. Addressing these blind spots requires high‐resolution tracking of EV interactions with defined human cell subsets and single‐cell analyses of downstream functional outcomes to distinguish between homeostatic recycling and maladaptive, stress‐induced signaling.

## 3 Discussion

The biology of hematopoietic aging has traditionally been interpreted through the lens of cell‐intrinsic alterations within HSCs, including telomere shortening, the accumulation of DNA damage, epigenetic drift, and reduced self‐renewal capacity. However, increasing evidence indicates that the aging phenotype of the hematopoietic system cannot be fully explained by intrinsic mechanisms alone. Instead, aging must be understood within the context of a dynamically evolving BM microenvironment in which EVs function as critical mediators of intercellular communication (Jeppesen et al. 2019; Kang et al. 2020; Ratajczak et al. 2006; Andaloussi et al. 2013; Valadi et al. 2007). EVs released by stromal cells, immune cells, and hematopoietic populations provide a mechanism through which molecular signals including proteins, lipids, and regulatory RNAs are transferred between niche components, influencing stem cell behavior and hematopoietic homeostasis. These studies position EVs as an additional regulatory layer in the aging BM niche, capable of coordinating systemic and local signals that shape HSC function over time (Batsali et al. 2020; Kfoury et al. 2021; Preciado et al. 2019).

In vitro mechanistic frameworks reveal a profound bidirectional and cross‐tissue reach of EV‐mediated aging signals. Within the localized murine bone marrow niche, cultured senescent macrophages, osteolineage cells, and damaged stromal cells demonstrate an increased release of pro‐senescent EVs. These vesicles are enriched in miR‐183‐5p, miR‐34a, and specific inflammatory proteins that directly impair murine HSC self‐renewal and skew differentiation toward myeloid fates (Davis et al. 2017). Concurrently, the reservoir of youthful, protective murine EVs which are rich in antioxidant enzymes and pro‐regenerative microRNAs progressively declines with age (Kulkarni et al. 2018; Liu et al. 2019). Furthermore, in vitro models demonstrate that this microenvironmental deterioration is driven not just by bone marrow‐resident cells, but also by peripheral tissues; for instance, EVs derived from aged murine skeletal muscle or cultured senescent macrophages actively induce secondary cellular stress when incubated with BM stromal fractions (Fulzele et al. 2019; Laliberte et al. 2024).

In human cellular in vitro systems, evidence highlights a non‐cell‐autonomous dimension of clonal dynamics and stem cell maintenance. Cultured MSCs from aged human donors secrete EVs enriched in miR‐29a and miR‐34a that significantly impair healthy HSPC homeostasis (Fichtel et al. 2022). These altered microenvironmental signals may disproportionately favor the survival and expansion of clones harboring specific age‐related mutations. Conversely, exposure to youthful human vesicle pools yields highly rejuvenative outcomes. EVs derived from young human plasma or healthy adult BMSCs significantly enhance the expansion, survival, and primitive stemness profiles of human HSPCs when co‐cultured in vitro (Ghebes et al. 2021; Grenier‐Pleau et al. 2020). Together, these findings suggest that clonal outcomes may depend less on individual EV miRNAs than on the overall balance of pro‐aging versus rejuvenating EV signals in the marrow niche.

Preclinical mouse models validate that age‐associated EV cargo remodeling operates at both local and systemic levels, driving macro‐environmental niche decline and providing avenues for intervention. Functional EV transfer experiments demonstrate that systemic administration of aged peripheral or tissue‐specific vesicles can replicate key features of hematologic aging in vivo, propagating chronic inflammatory signaling across organ systems and accelerating bone marrow microenvironmental decline (Fulzele et al. 2019) (Abbasi Sourki et al. 2023). Conversely, interventional studies using mouse models show that restoring a youthful systemic EV profile can counteract these phenotypes. Systemic delivery of young murine serum‐derived EVs effectively blunts systemic inflammaging, restores metabolic parameters within the BM cavity, and rescues aspects of immune cell fitness in vivo (Wang et al. 2018). However, several critical preclinical questions remain unaddressed. It is currently unknown whether exposure to aged EVs differentially expands mutant versus wild‐type HSC clones in competitive repopulation assays, or how age‐associated shifts in EV surface chemistry alter cell subset targeting specificity within the complex, three‐dimensional architecture of the aging bone marrow cavity. Side‐by‐side competitive repopulation experiments comparing the response of wild‐type and leukemia‐associated clones will be required to define the exact therapeutic window for engineered, niche‐targeted rejuvenative vesicles.

Observational studies of human cohorts across the lifespan establish that circulating EV cargo undergoes highly reproducible, age‐dependent changes that correlate closely with clinical stress states. Long‐term profiling reveals that the molecular payload of human plasma EVs mirrors systemic inflammatory burden and multi‐tissue aging (Mensa et al. 2020). Intriguingly, plasma EVs from individuals over 40 years old stimulate human HSC activity more robustly than those from young donors in vitro, a distinct cargo shift that is detectable well before clinically apparent clonal hematopoiesis of indeterminate potential (CHIP) or age‐related clonal hematopoiesis (ARCH) manifests (Grenier‐Pleau et al. 2020). These clinical correlations suggest that longitudinal plasma EV profiling could serve as an integrated, non‐invasive biomarker of multi‐tissue stress and an early warning readout for hematopoietic dysfunction, preceding overt clinical disease by years. Therapeutically, modulating this systemic signaling or restoring a youthful EV profile represents a promising clinical strategy to preserve human BM homeostasis, reduce the competitive advantage of mutated clones, and prospectively mitigate the progression of ARCH toward overt hematologic malignancy.

Finally, what is the therapeutic window for EV‐based rejuvenation strategies, and can engineered vesicles be directed with sufficient precision to both normal and mutant HSCs without broadly perturbing niche homeostasis? This question applies to both populations: restoring youthful EV signaling should ideally support normal HSC function thereby reducing the competitive advantage of mutant clones. Side‐by‐side competitive repopulation experiments comparing the response of wild‐type and leukemia‐associated clones will shed light on avenues to exploit candidate therapeutic EVs.

## 4 Conclusions and Future Directions

EVs have emerged as central coordinators of the aging BM niche, mediating a bidirectional shift toward pro‐senescent signaling that collectively accelerates hematopoietic aging across stromal, osteolineage, and immune niche populations. Whether aged niche‐derived EVs selectively amplify mutants over wild‐type HSC clones in aged populations is an open question that needs to be addressed. Competitive in vivo transplantation assays pairing fluorescently labeled aged HSCs against young counterparts, with aged versus young MSC‐derived EV exposure and cargo‐depletion strategies, would directly identify the responsible components, while single‐cell transcriptomic profiling would resolve downstream mechanisms. Additionally, the effect of EVs derived from aged or young HSC on the BM niche should also be addressed.

Prospective validation of plasma EV cargo as a biomarker of clonal emergence remains an open priority. Serial EV profiling in biobanks with linked hematopoiesis sequencing data should test whether EV surface composition or miRNA cargo shifts precede detectable clonal expansion.

Three‐dimensional culture systems remain underexplored for studying EV biology in the aging hematopoietic niche, yet proof‐of‐concept exists in adjacent systems: MSC‐derived EVs attenuate senescence in cholangioid organoids by reducing p16^INK4a^, p21^WAF1/Cip1^, and SASP components (Chen et al. 2021), and spheroid architecture itself shapes EV cargo composition and yield in ways that 2D culture cannot recapitulate (Rovere et al. 2023), together suggesting that BM‐specific 3D co‐culture systems incorporating aged stromal populations and HSCs represent a tractable next step for the field.

Finally, the translational potential of EVs in hematopoietic aging is twofold: whether youthful EVs can be developed as therapeutic vehicles to restore niche homeostasis, and whether the cargo shifts that characterize aged EVs can be harnessed as biomarkers of hematopoietic decline.

## Author Contributions

Kr.K. and Ka.K. conceptualized and supervised the review and provided funding. E.G.K., N.H., E.D., K.G. performed formal analysis, validation and data curation. E.G.K., N.H., E.D., K.G., Kr.K, Ka.K. wrote the original draft and reviewed edited the writing. N.H. visualized the figures.

## Funding

The study was funded by in whole or in part by European Hematology Association (BCG‐202209‐02649); in whole or in part by Blood Cancer UK (23001); in whole or in part by Mayo Clinic Robert and Arlene Kogod Center on Aging; Mayo Clinic Division of Hematology; Mayo Clinic Department of Internal Medicine; in whole or in part by the Austrian Science Fund (FWF) (Grant DOI: 10.55776/PIN1612324).

## Conflicts of Interest

The authors declare no conflicts of interest.

## Data Availability

Data sharing not applicable to this article as no datasets were generated or analysed during the current study.
